# Role of Innate Lymphoid Cells in Chronic Rhinosinusitis: Insights from Tissue and Peripheral Blood Flow Cytometric Analysis

**DOI:** 10.3390/arm94030035

**Published:** 2026-06-03

**Authors:** Rina Hoffmann, Franziska Rombach, Jens Grimm, Agmal Scherzad, Stephan Hackenberg, Pascal Ickrath

**Affiliations:** Department of Oto-Rhino-Laryngology, Head and Neck Surgery, University of Wuerzburg, 97074 Wuerzburg, Germany; ri.hoffmann@klinikum-stuttgart.de (R.H.); scherzad_a@ukw.de (A.S.);

**Keywords:** chronic rhinosinusitis with nasal polyps, chronic rhinosinusitis without nasal polyps, innate lymphoid cells

## Abstract

**Highlights:**

Innate lymphoid cells are key regulators of mucosal immunity in chronic rhinosinusitis, with ILC2s promoting type 2 inflammation in CRSwNP through IL-5 and IL-13 production, ILC3s supporting epithelial barrier defense via IL-17 and IL-22, and NK cells contributing to immune surveillance within the sinonasal tissue microenvironment. NK cell frequencies were reduced in CRSwNP nasal polyps but remained comparable between tissue and blood in CRSsNP, while ILCs in CRSwNP polyps showed increased GATA3 expression indicating local type 2 inflammation. Reduced frequencies of IL18Rα ILC2s, ILC3s, and NK cell subsets in CRSwNP polyps suggest a limited contribution of IL-18-mediated activation in local inflammation.

**What are the main findings?**

- Natural killer (NK) cells are very heterogenous and less mature in nasal tissues.
- Changes in the IL18Rα expression of ILC3s were found.

**What are the implications of the main findings?**

- NK cells appear to have a more prominent role in the pathophysiology of CRSNP.
- Results indicate a potential role of IL18 signaling in CRS pathogenesis.

**Abstract:**

(1) Background: Innate lymphoid cells (ILCs) are potent cytokine producers that regulate local immune responses in tissues. Natural killer (NK) cells belong to group 1 ILCs and play an important role in tumor clearance and defense against intracellular pathogens. ILC2 and 3 have been implied in allergic responses and other chronic inflammatory diseases. The role of these cells in the pathogenesis of chronic rhinosinusitis (CRS) is not completely understood. There are changes in the cellular infiltrate in the mucosa of patients with CRS with and without polyps. The aim of this study was to characterize the number and phenotype of NK cells, ILC2s and ILC3s in patients with CRS. (2) Methods: Tissue samples were collected from patients with CRS with and without nasal polyps who were undergoing nasal sinus surgery as well as control patients who were undergoing surgery due to non-inflammatory reasons. Lymphocytes were isolated from the tissues using mechanical and enzymatic dissociation. Peripheral blood lymphocytes were obtained from the same patients. All cells were examined by multicolor flow cytometry. NK cells were analyzed for the distribution of CD56^dim^CD16^+^ and CD56^bright^CD16^−^ subsets and the expression of IL18Rα, CD16, CD57, GATA3, TCF1 and NKp44. In ILC2s, GATA3 and IL18Rα expression was determined, and ILC3s as well as NKp44^+^ and NKp44^−^ILC3 subsets were analyzed for the expression of IL18Rα. (3) Results: There were significantly fewer NK cells in the nasal polyps compared to the peripheral blood of patients with CRSwNP and tissues from CRSsNP patients, which both showed higher levels of TCF1 expression. Irrespective of the disease condition, NK cells in tissues showed lower CD16 expression and a lower frequency of the CD56^dim^CD16^+^ subset compared to the peripheral blood mononuclear cells. Additionally, a smaller percentage of NK cells were terminally matured, as measured by CD16^+^ and CD57^+^ expression, in all examined nasal mucosa tissues. In the tissue ILC3s, we predominantly found cells from the NKp44^−^ subset in all groups. ILC3s from CRSsNP patients showed the highest frequencies of IL18Rα^+^ cells of all examined tissues. ILC2s from the polyps ofCRSwNP patients showed higher levels of GATA3 expression than their peripheral blood counterparts. (4) Conclusions: We found that tissue-resident NK cells in mucosa from the nose and sinuses are a more heterogenous and less mature population than those in peripheral blood. Expression of the examined markers in NK cells was similar among groups. NK cell frequency, both in blood and tissue from CRSsNP patients, was higher than in the other groups, indicating that these cells might play an important role in this phenotype. Changes in the IL18Rα expression of ILC3s suggest a potential role of IL18 signaling in CRS pathogenesis.

## 1. Introduction

Chronic rhinosinusitis is an inflammatory disease of the nasal mucosa (rhinitis) and of the paranasal sinus mucosa (sinusitis). The worldwide prevalence varies, and in Europe, it is estimated to be at about 6–15% [1]. According to the frequencies of symptoms and their duration, there are three different clinically known forms: acute rhinosinusitis (ARS), recurring acute rhinosinusitis (RARS) and chronic rhinosinusitis (CRS). CRS can be further divided according to its phenotype into chronic rhinosinusitis with nasal polyps (CRSwNP) and chronic rhinosinusitis without nasal polyps (CRSsNP). Common symptoms of CRS both with and without nasal polyps include nasal obstruction, anterior or posterior nasal drip, and tension headache or cephalgia as well as hyposmia or anosmia. Endoscopic swelling of the mucosa and purulent secretion [1] can be found in both phenotypes. In addition, the presence of polyps is obligatory in CRSwNP. Even though significant progress has been made in recent years regarding the understanding of the pathomechanism of CRS with the introduction of endotypes [2], many uncertainties still persist. Theories include allergic diathesis and chronic inflammatory reaction as well as anatomic variations. Dysfunctional epithelial resistance with acanthosis and acantholysis [3,4] is observed but currently not fully understood.

The immune system’s involvement in CRS pathophysiology is significant, particularly through the activation of the innate immune system, which detects unique molecular structures of microorganisms. Key components include phagocytes, mast cells, granulocytes, natural killer (NK) cells, and other innate lymphoid cells (ILCs). ILCs, including ILC2, ILC3, and NK cells, play vital roles in various immune responses during acute infection and chronic inflammation. In recent years, numerous studies have demonstrated that ILC2s play a central role in type 2 inflammatory responses by producing cytokines such as IL-5 and IL-13, thereby promoting eosinophilic inflammation, mucus production, and epithelial barrier remodeling in allergic diseases including asthma and chronic rhinosinusitis [5]. ILC3s produce cytokines like IL-5 and IL-13 and contribute to airway hyperresponsiveness, mucus production and eosinophilic inflammation. Both ILC2 and ILC3 cells appear to be involved in the inflammation seen in CRSwNP. While ILC2s drive type 2 inflammation, ILC3s have been suggested to contribute to tissue remodeling and chronic inflammation.

ILC2s, characterized by high GATA3 expression, are implicated in increased mucus production and eosinophilia in airways, often observed in conditions like allergic rhinitis and CRSsNP. Similarly, ILC3s, differentiated by expression of retinoic acid-related orphan receptor gamma t (RORɣt), contribute to immune responses against bacterial infections and intestinal homeostasis.

NK cells, a subset of type 1 ILCs, exert cytotoxic activity against virus-infected and transformed epithelial cells and contribute to mucosal immune surveillance in the upper airways. By eliminating infected or damaged epithelial cells, they help regulate pathogen-driven inflammation and maintain epithelial barrier integrity. Increased frequencies of NK cells in nasal polyps compared with peripheral blood and nasal mucosa suggest a role in the local inflammatory processes of CRS [6]. NK cell subsets, characterized by CD56^+^ CD3 expression, are found in various tissues with different functional properties. In CRS patients, higher frequencies of NK cells are found in polyps compared to peripheral blood and nasal mucosa.

Cellular markers such as NKp44, IL18R, GATA3, TCF1 and CD57 play important roles in immune cell activation, differentiation, and cytokine production, with implications for diseases like CRS and autoimmune conditions [7,8,9]. Understanding the roles of innate immune cells and associated markers provides insights into CRS pathogenesis and potential therapeutic targets. Many studies showed an increased concentration of immune cells in the inflamed nasal mucosa [10,11] but were limited in the number of cell type markers used as well as focused mostly on peripheral blood samples. Until now there has been no study comparing paired tissue and blood samples of patients from these different groups. The aim of the present study was to identify potential differences between tissue and blood samples among the various groups.

Our hypothesis is that tissue-resident ILCs play a significant role in maintaining the local epithelial inflammation in CRS.

## 2. Materials and Methods

### 2.1. Ethical Issues

All participants of this study gave written informed consent. The study was approved by the Ethics Board of the Medical Faculty, Julius-Maximilian-University Wuerzburg (No. 116/17).

### 2.2. Preparation of Peripheral Blood Mononuclear Cells

To isolate peripheral blood mononuclear cells (PBMCs), 18 mL of heparinized blood was collected from the participants on the day of surgery by venous puncture. Separation of PBMCs was done using density-gradient centrifugation (10 min, 1000× *g*) at room temperature (RT) on equal amounts of Ficoll (Biochrom, Berlin, Germany) in a 50 mL cell tube (Greiner Bio-One, Frickenhausen, Germany). To determine cell count and viability, a Cell Counter + Analyzer System (CASY TT, Innovatis, and Reutlingen) was used according to the manufacturer’s protocol. Cells were washed with PBS and centrifuged (500× *g*). Prior to analysis, cells were frozen at −80 °C for 12 h and at −190 °C in liquid nitrogen until further use. Freezing medium (1 mL/sample) was added which was prepared using 10 parts of fetal calf serum (LINARIS, Dossenheim, Germany) and one part of DMSO (Roth, Karlsruhe, Germany).

### 2.3. Preparation of Tissue Samples

Tissue samples were collected from CRSwNP (*n* = 11) and CRSsNP (*n* = 10) patients as well as patients without CRS who had nasal surgery due to non-inflammatory reasons (CTRL *n* = 7). Dissociation was done using the Multi Tissue Dissociation Kit 1 and the gentleMACSDissociator with the program Multi A (Miltenyi Biotec GmbH, Bergisch Gladbach, Germany) according to the manufacturer’s protocol. The suspension was filtered through a mesh (sized 100 to 40 µm) and the tubes were washed with 15 mL RPMI solution (Biochrom GmbH, Berlin, Germany). Prior to analysis, the cells were counted and stored frozen as described above.

### 2.4. Fluorescence-Activated Cell Sorting

Characterization of the ILCs was done by fluorescence-activated cell sorting (FACS). The antibodies were used according to the manufacturer’s protocol. Cells were stained with Viability Dye 780 (eBioscience; Thermo Fisher Scientific Inc., Waltham, MA, USA) at 1:1000 and FcR blocking was performed using an FcR blocking reagent (Miltenyi Biotec GmbH, Bergisch Gladbach, Germany) at 1:10 in FACS buffer (phosphate-buffered saline (PBS, Roche Diagnostics GmbH, Rotkreuz, Switzerland), 0.5% bovine serum albumin (BSA, Carl Roth GmbH, Karlsruhe, Germany), 2 mM EDTA (Sigma-Aldrich, St. Louis, MO, USA)) for 20 min on ice. Lineage staining was performed in FACS buffer for 20 min on ice. For surface staining, Streptavidin V500 (BD Biosciences, Franklin Lakes, NJ, USA) was added to the antibody panel and the samples were incubated for 10 min at 37 °C on a shaker, followed by 15 min at 4 °C. After fixation with fixation buffer (eBioscience; Thermo Fisher Scientific Inc., Waltham, MA, USA), whole-cell staining was performed in permeabilization buffer (eBioscience; Thermo Fisher Scientific Inc., Waltham, MA, USA) for 30 min at 4 °C. For each group a small sample was left without staining in the FITC channel as a fluorescence-minus-one (FMO) control. FACS analysis was performed using aCytoFLEX Flow Cytometer LX (Beckman Coulter, Brea, CA, USA). A compensation was calculated with the VersaComp Antibody Capture Kit (Beckman Coulter, Brea, CA, USA). For data analysis, the FlowJo 10.2 software (FlowJo LLC, Ashland, OR, USA) was used.

### 2.5. Gating Strategy

**Figure 1:**
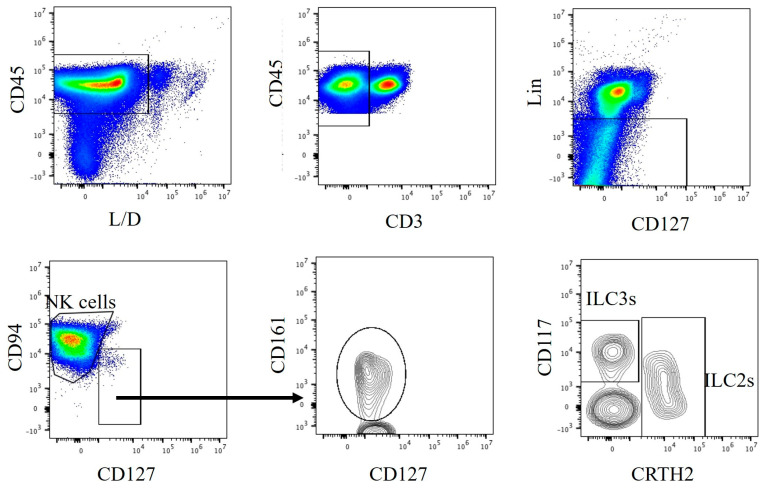
Gating strategy for identification of NK cells, ILC2s and ILC3s in multicolor flow cytometry. The first two gates were set to gate out doublets and to focus on the population of interest (not displayed). Lineage markers (Lins) included BDCA-2, CD1a, CD4, CD5, Cd14, CD19, CD20, CD34, CD123, FcR1, TCRβ, and TCRγ. Lymphocytes were live/dead L/D^−^ and CD45^+^. Cells that were L/D^−^, CD45^+^, CD3^−^, Lin^−^, CD94^+^, or CD127^−^ were categorized as NK cells. ILC2s and ILC3s were identified by L/D^−^, CD45^+^, CD3^−^, Lin^−^, CD94^−^, CD127^+^, or CD161^+^ expression and distinguished from one another by CRTH2 expression (ILC3s: CRTH2^−^; ILC2s: CRTH2^+^).

The gating strategy is depicted in Figure 1. After gating out doublets, a gate was set on the population of interest. Dead and apoptotic cells (live/dead (L/D)-positive) were excluded and lymphocytes were identified by CD45 expression. Lineage markers included BDCA-2, CD1a, CD4, CD5, Cd14, CD19, CD20, CD34, CD123, FcR1, TCRβ and TCRγ. In the lineage (Lin)-negative cells, NK cells were distinguished from other ILCs by CD94 and CD127 expression. CD161^+^ cells were examined for their CD117 and CRTH2 expression to identify ILC3s (CRTH2^−^) and ILC2s (CRTH2^+^).

### 2.6. Statistics

Data are presented as means ± standard error of the mean (SEM). For comparison of the mean fluorescence intensity (MFI), the relative MFI (rMFI) was calculated by normalization of sample values to the values of the FMO control. Statistical significance was tested by Two-way ANOVA with Tuckey’s multiple comparison test using the software Prism 6.0c (GraphPad by Dotmatics, Boston, MA, USA). Values of *p* < 0.05 were deemed statistically significant.

## 3. Results

### 3.1. Patient Characteristics

A total of 28 patients were included in the study. For a summary of patient characteristics, see Table 1.

The surgeries were performed at the Department of Otorhinolaryngology, Plastic, Aesthetic and Reconstructive Head and Neck Surgery of the University of Wuerzburg (Wuerzburg, Germany). Causes for surgery on control patients included defects of the skull base (4), deviation of the nasal septum (1), concha bullosa (1) and a choanal polyp (1). All patients received conservative therapy prior to surgical treatment. In cases of non-response to conservative therapy, the indication for paranasal sinus surgery was provided according to the European guidelines [1]. Based on pre- and intraoperative and endoscopic as well as radiological findings, patients were classified into CRSwNP and CRSsNP subgroups. Patients with primary ciliary dyskinesia, eosinophilic granulomatosis with polyangiitis, or cystic fibrosis were excluded. All participants were enrolled in the study at the Department of Otorhinolaryngology, Plastic, Aesthetic, and Reconstructive Head and Neck Surgery of the University of Wuerzburg (Wuerzburg, Germany).

### 3.2. Frequency of Tissue-Associated ILCs

**Figure 2:**
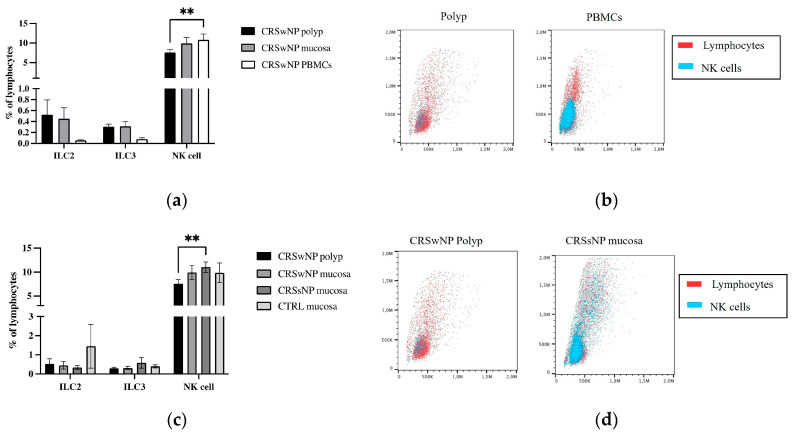
(**a**) Frequencies of ILC2s, ILC3s and NK cells in patients with CRSwNP in tissue-resident lymphocytes and lymphocytes of the peripheral blood. There were significantly fewer NK cells in the polyp compared to the peripheral blood (*p* = 0.0026). The percentage of ILC2s in the polyp and mucosa was up to 10× higher than that in the peripheral blood. This difference was not significant. (**b**) Example for NK cell frequencies in the polyp compared to the peripheral blood. (**c**) Frequencies of ILC2s, ILC3s and NK cells in tissue-resident lymphocytes of patients with CRSwNP and CRSsNP and CTRL patients. There were significantly more NK cells in the mucosa of CRSsNP patients compared to the polyp in CRSwNP patients (*p* = 0.0057). (**d**) Example for NK cell frequencies in the polyp from CRSwNP compared to CRSsNP tissue. ** *p* ≤ 0.005.

The frequencies of ILC2s, ILC3s and NK cells among all lymphocytes (CD45^+^) were measured. In CRSwNP patients, ILC2s comprised 0.53% of lymphocytes in the polyp tissue, 0.45% in the control mucosa and 0.058% in the PBMCs (see Figure 2a). Although the percentage of tissue-resident ILCs was up to ten times higher than that in PBMCs, this difference between tissues, polyps, and blood was not statistically significant. There was a higher percentage of ILC3s in the tissues with 0.30% of lymphocytes in the polyps, 0.31% in the mucosa and 0.79% in the peripheral blood. NK cells were significantly more abundant with 10.82% in PBMCs (*p* = 0.0026) compared to the polyp with 7.59 +/−% (*p* = 0.0026, see Figure 2a,b). In the mucosa, 9.94% of lymphocytes were NK cells.

Compared to the other groups, the frequency of tissue-resident NK cells was lowest in the polyps from CRSwNP patients and significantly lower than in those from CRSsNP patients, where they accounted for 11.04% of lymphocytes (*p* = 0.0057, see Figure 2a–d). In CTRL participants, 9.87% of lymphocytes were NK cells. The frequency of tissue-resident ILC2s and ILC3s did not differ among groups with 0.34% (CRSsNP) and 1.45% (CTRL) for ILC2s and 0.59% (CRSsNP) and 0.40% (CTRL) for ILC3s (see Figure 2a,c).

NK cells in the nasal mucosa are a more heterogenous and less mature population compared to those in the peripheral blood.

### 3.3. CD16 Expression of Tissue-Resident NK Cells

**Figure 3:**
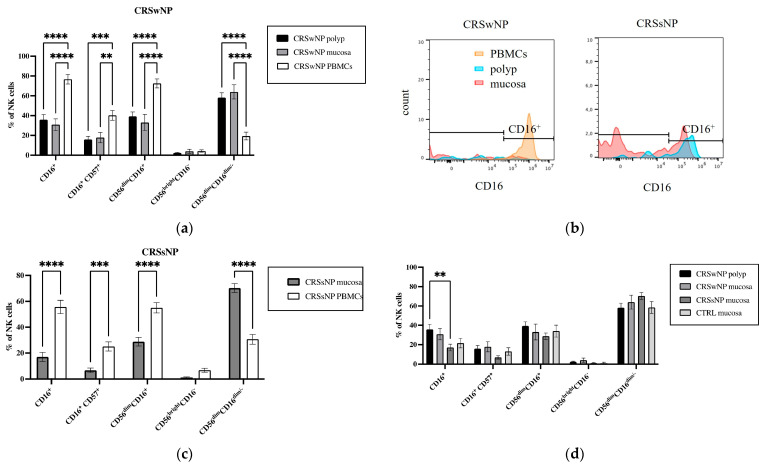

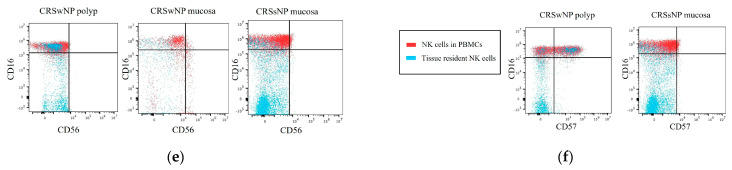
NK cells in CRSwNP (**a**) and CRSsNP (**c**) patients and all tissues (**d**). Frequency of CD16^+^, CD16^+^ CD57^+^, CD56^dim^ CD16^+^, CD56^bright^ CD16^−^, and CD56^dim^ CD16^−^ cells. There were significantly more CD16^+^, CD16^+^ CD57^+^, and CD56^dim^ CD16^+^ cells in the peripheral blood compared to tissue-resident NK cells from the tissues of CRSwNP and CRSsNP patients (** *p* < 0.005, *** *p* < 0.0005, **** *p* < 0.0001). A significantly higher frequency of NK cells were of the CD56^dim^ CD16^−^ subset in the tissues from CRSsNP and CRSwNP patients compared to the peripheral blood (**** *p* < 0.0001). Subset distribution and expression of CD16 and CD57 did not differ significantly among tissue-resident NK cells of all groups (**d**). (**b**) Example of CD16 expression in CRSwNP and CRSsNP patients. (**e**) Subset distribution by CD16 and CD56 expression of tissue-resident NK cells compared to that in the peripheral blood in CRSsNP and CRSwNP patients. (**f**) CD16 and CD57 expression in the mucosa from CRSsNP as well as the polyp from CRSwNP patients compared to that in peripheral blood NK cells.

Flow cytometric analysis of tissue-resident NK cells showed a lower expression of CD16 in the tissues with fewer **CD16^+^CD57^+^** cells. In CRSwNP patients, 35.81% (polyp) and 30.78% (mucosa) of tissue-resident NK cells were CD16^+^ compared to 76.68% in the peripheral blood (*p* < 0.0001, see Figure 3a,b). In peripheral blood samples of CRSwNP patients, significantly more cells (72.55%) were of the **CD56^dim^CD16^+^** phenotype compared to the tissue-resident NK cells (polyp: 39.30%, *p* < 0.0001; mucosa: 33.08%, *p* < 0.0001; see Figure 3a). There was also a higher frequency of the **CD56^bright^CD16^−^** subset (4.22% in PBMCs, 2.27% in the polyp and 3.94% in the mucosa) but this difference did not reach the level of significance. There were multiple levels of CD16 expression in the tissues and tissue-resident NK cells were predominantly **CD56^dim^CD16^−^** (58.18% in the polyp, 64.00% in the mucosa; see Figure 3a,d). This was a significantly higher frequency than that in the peripheral blood (19.36%, *p* < 0.0001). The percentage of terminally matured **CD16^+^CD57^+^** cells was higher in peripheral blood (40.28%) than in polyps (15.80%, *p* = 0.0001; see Figure 3a) and mucosa (17.79%, *p* = 0.0062).

In CRSsNP patients, 17.02% of tissue-resident cells were CD16^+^. Peripheral blood NK cells showed higher levels of CD16 expression with 55.68% CD16^+^cells (*p* < 0.0001, see Figure 3b,c). Analysis of the subset distribution showed 55.06% were of the **CD56^dim^CD16^+^** subset and 6.83% of the **CD56^bright^CD16^−^** subset in PBMCs. In the tissue, fewer of the cells belonged to these subsets with 28.79% for the **CD56^dim^CD16^+^** (*p* < 0.0001) and 1.149% for the **CD56^bright^CD16^−^** (n.s., see Figure 3c,e) subset. The majority (70.34%) of NK cells were **CD56^dim^CD16^−^** while only 30.65% of NK cells in peripheral blood showed a similar expression profile (*p* < 0.0001, see Figure 3c,e). As observed in the polyps, fewer of the tissue-resident NK cells (6.80%) were terminally matured (**CD16^+^CD57^+^**) as compared to the peripheral blood NK cells (25.09%, *p* = 0.0008; see Figure 3c,f).

Comparison of all tissue-resident NK cells showed a significantly higher frequency of CD16^+^ NK cells in CRSsNP tissues compared to polyps from CRSwNP patients (*p* = 0.0023, see Figure 3b,d). No further significant differences in CD16, CD56 and CD57 expression among the study groups (see Figure 3d) were found. In CTRL participants, 21.62% of NK cells were CD16^+^. **CD16^+^CD57^+^** cells made up 12.96% of all NK cells and subset distribution was measured at 34.01% for the **CD56^dim^CD16^+^** subset, 1.06% for the **CD56^bright^CD16^−^** subset and 58.44% for **CD56^dim^CD16^−^** cells.

Isolated NK cells from nasal polyps show higher levels of TCF1/7 expression compared to tissue-resident NK cells from the other groups.

### 3.4. Levels of GATA3 and TCF1/7 and Frequencies of Nkp44^+^ and IL18Rα^+^ Cells

**Figure 4:**
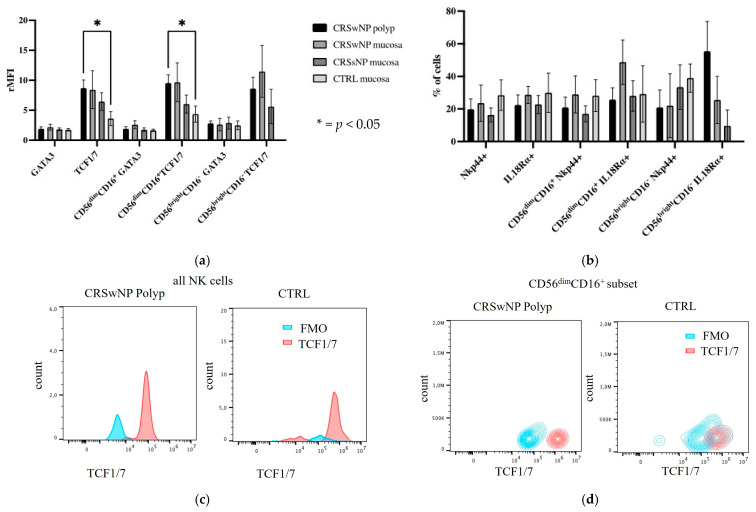
(**a**) Expression of GATA3 and TCF1/7 in NK cells and NK cell subsets in tissue-resident cells from CRSwNP and CRSsNP patients as well as CTRLs. TCF1/7 expression was higher in the polyp compared to tissue from CTRLs in all NK cells ((**c**) *p* = 0.031) and in the CD56^dim^ CD16^+^ subset ((**d**) *p* = 0.028). There was no significant difference in the level of GATA3 expression among groups. (**b**) Expression of Nkp44 and IL18Rα in tissue-resident NK cells from CRSwNP and CRSsNP patients as well as CTRLs. The expression of these markers was similar among groups. * = *p* < 0.05.

To determine the activation status of tissue-resident NK cells, we examined the expression levels of GATA3 and TCF1/7 as well as the frequencies of Nkp44^+^ and IL18Rα^+^ cells. There was no difference in the level of **GATA3** expression with rMFIs of 1.93 (CRSwNP polyp), 2.18 (CRSwNP mucosa), 1.84 (CRSsNP mucosa) and 1.76 (CTRL mucosa) in all NK cells; 1.93 (CRSwNP polyp), 2.56 (CRSwNP mucosa), 1.76 (CRSsNP mucosa) and 1.68 (CTRL mucosa) in the CD56^dim^CD16^+^ subset; and 2.81 (CRSwNP polyp), 2.64 (CRSwNP mucosa), 2.87 (CRSsNP mucosa) and 2.49 (CTRL mucosa) in the CD56^bright^CD16^−^ subset (see Figure 4a). Tissue-resident NK cells from CRSwNP patients showed the highest levels of **TCF1/7** expression with rMFIs of 8.73 (polyp) and 8.44 (mucosa) for all NK cells; 9.55 (polyp) and 9.65 (mucosa) for the CD56^dim^CD16^+^ NK cells; and 8.65 (polyp) and 11.50 (mucosa) for the CD56^bright^CD16^−^ subset (see Figure 4a,c,d). NK cells from CTRL tissue had significantly lower levels of **TCF1/7** expression than cells from the polyp with rMFIs calculated at 3.62 (all NK cells, *p* = 0.031; see Figure 4a,c); 4.37 (CD56^dim^CD16^+^ subset, *p* = 0.028; see Figure 4a,d) and 0.016 (CD56^bright^CD16^−^ cells, data from only one patient, n.s.). In CRSsNP, the rMFIs of TCF1/7 were 6.46 (all NK cells); 6.05 (CD56^dim^CD16^+^ subset) and 5.64 (CD56^bright^CD16^−^ cells). 

Expression of Nkp44 and IL18Rα was similar among groups (see Figure 4b). In all NK cells, frequencies of **Nkp44^+^** cells were 19.88% (CRSwNP polyp), 23.61% (CRSwNP mucosa), 16.40% (CRSsNP mucosa) and 28.51% (CTRL mucosa). In CD56^dim^CD16^+^ NK cells, 20.97% (CRSwNP polyp), 29.00% (CRSwNP mucosa), 17.03% (CRSsNP mucosa) and 28.24% (CTRL mucosa) were Nkp44^+^ while in the cytokine-producing subset (CD56^bright^CD16^−^), 21.10% (CRSwNP polyp), 22.00% (CRSwNP mucosa), 33.39% (CRSsNP mucosa) and 28.97% (CTRL mucosa) of cells expressed Nkp44. **IL18Rα** expression was measured in 22.50% (CRSwNP polyp), 28.68% (CRSwNP mucosa), 22.75% (CRSsNP mucosa) and 30.00% (CTRL mucosa) of NK cells. In the CD56^dim^CD16^+^ subset the frequencies of IL18Rα-expressing cells were 25.82% (CRSwNP polyp), 48.74% (CRSwNP, mucosa), 28.02% (CRSsNP mucosa) and 29.22% (CTRL mucosa). CD56^bright^CD16^−^ cells showed expression rates of 55.42% (CRSwNP polyp), 22.55% (CRSwNP mucosa), 9.70% (CRSsNP mucosa) and 0% (CTRL mucosa, data from only one patient). 

ILC2s from patients with CRSwNP show higher levels of GATA3 expression in nasal polyps than in peripheral blood.

**Figure 5:**
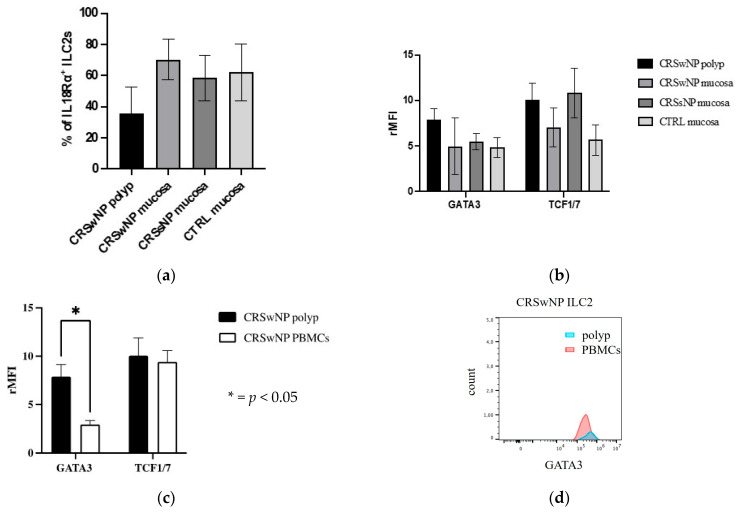
(**a**) Expression of IL18Rα, (**b**) GATA3 and TCF1/7 in tissue-resident ILC2s from patients with CRSwNP and CRSsNP as well as CTRLs. There was no significant difference in the expression of these markers. (**c**) Expression of GATA3 and TCF1/7 in ILC2s from CRSwNP patients. The level of GATA3 expression was significantly higher in the polyp compared to the peripheral blood (*p* = 0.045). (**d**) GATA3 expression in isolated polyp and peripheral blood ILC2s from CRSwNP patients. * = *p* < 0.05.

The frequency of **IL18Rα^+^** ILC2s was not significantly different among all examined tissues (see Figure 5a): 35.55% (polyp) and 70.27% (mucosa) in CRSwNP patients, 58.30% in CRSsNP patients and 61.96% in the CTRL mucosa. The highest **GATA3** expression was measured in tissue-resident ILC2s from nasal polyps in CRSwNP patients (rMFIs of 7.91 in nasal polyps, 4.99 in nasal mucosa). In CRSsNP tissues the rMFI of **GATA3** was 5.48 and in CTRL mucosa was 4.84. **TCF1/7** expression was measured at 10.07 (polyp) and 7.04 (mucosa) in CRSwNP tissues, 10.87 in CRSsNP tissues and 5.69 in the CTRL ILC2s (see Figure 5b). ILC2s isolated from nasal polyps showed significantly higher rMFIs for **GATA3** than ILC2s from peripheral blood (rMFI: 2.95, *p* = 0.045; see Figure 5c,d), while the rMFI of **TCF1/7** was similar in these cells (rMFI in CRSwNP PBMCs: 9.40).

### 3.5. Tissue-Resident ILC3s in the Nasal Mucosa Are Predominantly of the Nkp44^−^ Subset

**Figure 6:**
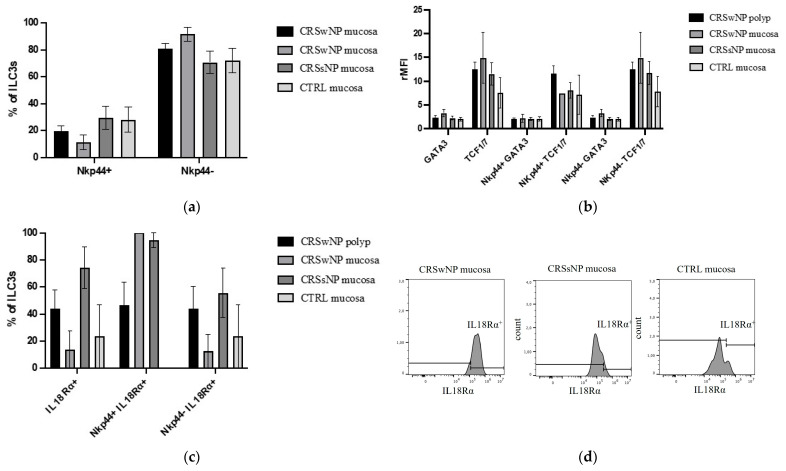
(**a**) Distribution of Nkp44^+^ and Nkp44^−^ subsets in tissue-resident ILC3s from CRSwNP and CRSsNP patients as well as CTRLs. The cells were predominantly Nkp44^−^ ILC3s in all tissues. (**b**) Expression of IL18Rα in tissue-resident ILC3s and subsets from patients with CRSwNP and CRSsNP as well as CTRLs. ILC3s from CRSsNP patients showed the highest IL18Rα expression rate (**d**), but this difference was not significant. (**c**) Expression of GATA3 and TCF1/7 in ILC3s from CRSwNP and CRSsNP patients as well as CTRLs.

Tissue-resident ILC3s in all examined tissues were predominantly of the **Nkp44^−^** subset (see Figure 6a). In the CRSwNP patients, 80.89% (polyp) and 91.69% (mucosa) of ILC3s were **Nkp44^−^**. In CRSsNP and CTRL mucosa, the frequencies of this subset were 70.64% (CRSsNP) and 71.97% (CTRL). As displayed in Figure 6b, the expression of **GATA3** and **TCF1/7** did not differ among tissues and subsets. rMFIs for **GATA3** were 2.38 (all ILC3s), 2.11 (Nkp44^+^ subset) and 2.41 (Nkp44^−^ subset) in the polyps (CRSwNP); 3.32 (all ILC3s), 2.19 (Nkp44^+^ subset) and 3.31 (Nkp44^−^ subset) in CRSwNP mucosa; 2.28 (all ILC3s), 2.13 (Nkp44^+^ subset) and 2.07 (Nkp44^−^ subset) in CRSsNP mucosa and 2.05 (all ILC3s), 2.12 (Nkp44^+^ subset) and 208 (Nkp44^−^ subset) in CTRL mucosa. For **TCF1/7** the rMFIs were 12.51 (all ILC3s), 11.62 (Nkp44^+^ subset) and 12.56 (Nkp44^−^ subset) in the polyps (CRSwNP); 14.92 (all ILC3s), 7.46 (Nkp44^+^ subset, data from only one patient) and 14.92 (Nkp44^−^ subset) in CRSwNP mucosa; 11.53 (all ILC3s), 8.09 (Nkp44^+^ subset) and 11.75 (Nkp44^−^ subset) in CRSsNP mucosa and 7.58 (all ILC3s), 7.22 (Nkp44^+^ subset) and 7.86 (Nkp44^−^ subset) in CTRL mucosa.

The frequency of **IL18Rα^+^** ILC3s was highest in CRSsNP mucosa (n.s.) where 74.12% of all cells, 94.66% of Nkp44^+^ ILC3s and 55.58% of the Nkp44^−^ subset expressed the receptor (see Figure 6c,d). In CRSwNP patients, the expression rates were 43.73% (polyp) and 13.90% (mucosa), for all ILC3s; 46.53% (polyp) and 100% (mucosa, data from only one patient) for Nkp44^+^ ILC3s; and 44.08% (polyp) and 12.50% (mucosa) for the Nkp44^−^ subset. ILC3s isolated from CTRL tissues showed IL18Rα expression in 23.53% (all ILC3s), 0% (Nkp44^+^ subset, data from only one patient) and 23.53% (Nkp44^−^ subset) of the examined cells.

Table 2 summarizes selected markers across the individual study groups to provide a clearer overview.

## 4. Discussion

Only a few studies have characterized ILC2s, ILC3s and NK cells isolated from paranasal sinus tissue in patients with different subphenotypes of CRS. To determine specific properties of the ILCs in inflamed nasal mucosa, we isolated lymphocytes from healthy mucosa controls. We were able to show that the frequency of ILC2s, ILC3s and NK cells in the CTRL mucosa were similar to that in peripheral blood in healthy controls. As previously described by other groups, we also characterized tissue-resident NK cells as less mature [12] regarding CD16 expression compared to peripheral blood samples. We found a significantly lower frequency of CD16^bright^ but significantly more NK cells of other less mature stages (CD56^dim^CD16^dim^/-). The expression of Nkp44 in tissue-resident cells resembled the frequency of Nkp44^+^ cells in peripheral blood, indicating that tissue-resident NK cells in healthy mucosa, though less mature, appear to be in a similar state of activation. As previously described by other groups [13,14,15], we also found that tissue-resident and peripheral blood ILC3s were predominantly of the Nkp44^−^ subset. There were no differences in the expression of GATA3, IL18R and TCF1/7 in ILC2s and ILC3s in healthy tissue compared to that in peripheral blood, possibly due to low cell counts and low frequencies of ILC2s and ILC3s in non-inflamed tissues.

Previous research indicated higher NK cell abundance in inflamed tissue compared to peripheral blood [14], but the current study found a lower prevalence of NK cells in nasal polyp tissues compared to peripheral blood. This discrepancy may be explained by differences in tissue processing and lymphocyte isolation methods. In addition, the inflammatory microenvironment in nasal polyps may favor the recruitment or expansion of other immune cell populations, thereby reducing the relative proportion of NK cells. In healthy individuals, NK cells typically comprise 5–20% [16] of lymphocytes in nasal tissue. While NK cell frequencies in nasal tissue from CRSsNP resembled those in peripheral blood (11.04%), in CRSwNP patients, NK cell frequencies were lower in the nasal polyp tissue compared to peripheral blood. This suggests that other immune cells may drive the inflammation in CRSwNP. As described by previous groups [14], we also detected higher frequencies of both ILC2s and ILC3s in the nasal polyps than in the peripheral blood of patients with CRSwNP. These differences were not significant in this study and need to be confirmed with larger data collection.

Recent studies have highlighted the important role of innate lymphoid cells in the pathogenesis of chronic rhinosinusitis, particularly in CRSwNP, where ILC2s contribute substantially to type 2-driven inflammation within nasal polyp tissue [17,18]. Consistent with these findings, our data demonstrate increased transcriptional activity of type 2-associated pathways in tissue-resident ILC2s from nasal polyps, reflected by elevated GATA3 expression.

Soklic et al. [19] distinguished eosinophilic and non-eosinophilic CRSwNP patients while we did not differentiate between endotypes in the analysis of polyps in this study. This could explain the lack of significant GATA3 overexpression in tissue-resident lymphocytes as a marker for type 2 inflammation [14]. We found significantly lower frequencies of IL18Rα^+^ ILC2s in nasal polyps compared to PBMCs in patients with CRSwNP. In nasal mucosa of CRSwNP patients, lower percentages of IL18Rα-expressing cells were observed among the total amount of ILC3s and Nkp44^−^ ILC3s as well as CD56^bright^CD16^−^ NK cells, suggesting that IL18 signaling may not be upregulated in CRSwNP inflammation. IL18 signaling is regulated by many other factors, e.g., IL18 binding protein, and cannot only be measured by determination of IL18Rα levels. Further studies determining IL18 binding protein levels and IL18Rβ expression are needed to understand the role of IL18 in the pathogenesis of CRSwNP. To evaluate whether NK cells in polyps and nasal mucosa from patients with CRSwNP were more activated than in the peripheral blood, we measured Nkp44 expression. This member of the NCRs is only expressed in activated NK cells [20]. The frequency of Nkp44^+^ NK cells was similar in the tissue and blood of CRSwNP patients. This also applied to the CD56^dim^CD16^+^ and the CD56^bright^CD16^−^subsets in these patients. These results indicate that increased NK cell activation and Nkp44 overexpression are not involved in maintaining hyperinflammation in CRSwNP. Overall, our findings highlight the complex role of NK cells in CRSwNP inflammation and the need for further investigation into their specific functions within the nasal tissue microenvironment.

In contrast to our findings from CRSwNP patients, we could not find a decreased share of NK cells in tissue from patients with CRSsNP compared to the peripheral blood. Similarly to our findings in CRSwNP mucosa and findings of other groups [14], ILC2s and ILC3s seemed to be more abundant in the inflamed tissue than in the peripheral blood, but this difference was not statistically significant. Interestingly we found that the frequency of NK cells was significantly higher in the mucosa from CRSsNP patients than in polypoid tissue from CRSwNP patients. The percentage in CRSsNP tissue resembled NK cell frequencies in the peripheral blood. This indicates that NK cells play a bigger role in the pathogenesis of CRSsNP. Further characterization of tissue ILC2s in CRSsNP showed no difference in the expression of TCF1/7 and IL18Rα compared to ILC2s in PBMCs. Type 2 inflammation is scarcely found in the CRSsNP phenotype [2]. In line with this, GATA3 overexpression as observed in ILC2s from polypoid tissue in CRSwNP patients was not found in the CRSsNP group. There were also no significant differences in Nkp44 expression between tissue-resident cells and peripheral blood NK cells in CRSsNP patients, contrary to the findings in CRSwNP patients.

The reduced CD16 and CD57 expression observed in tissue-resident NK cells may indicate a less mature phenotype influenced by the local inflammatory milieu. Furthermore, the increased expression of transcription factors such as TCF1/7 in NK cells from CRSwNP tissues and the elevated GATA3 levels in ILC2s from nasal polyps may reflect tissue-specific activation and differentiation pathways driven by the local cytokine environment. In addition, emerging evidence suggests that the local tissue microenvironment plays a critical role in shaping the phenotype and functional properties of innate lymphoid cells and NK cells at mucosal barrier sites [21]. This concept may explain the phenotypic differences observed in our study between circulating PBMC-derived cells and tissue-resident NK cells and ILC subsets in sinonasal tissues.

Furthermore, recent work has demonstrated that the distribution and activation state of innate lymphoid cells may influence disease severity and therapeutic responses in CRSwNP [22]. Our findings of altered NK cell maturation markers and increased TCF1/7 expression in tissue-resident NK cells from CRSwNP patients may therefore reflect local immune adaptation within the inflammatory microenvironment of nasal polyps.

Finally, the predominance of specific ILC subsets within mucosal tissues has been linked to the broader inflammatory endotypes of chronic rhinosinusitis [23]. Recent studies further indicate that distinct ILC subsets and activation states exist within nasal polyp tissue, reflecting disease heterogeneity and emphasizing the importance of tissue-resident immune responses in CRSwNP pathophysiology [24]. In line with this concept, the distribution patterns of ILC2 and ILC3 populations observed in our study further support the idea that innate lymphoid cells contribute to shaping the local immune landscape in CRS.

This study has several limitations. Although both tissue samples and peripheral blood were analyzed, flow cytometric phenotyping alone cannot fully capture the complexity of the sinonasal tissue microenvironment. In addition, the relatively small sample size, and the lack of stratification of CRSwNP patients into inflammatory endotypes, may have limited the detection of subtle differences between groups. Future studies including larger cohorts and functional analyses are needed to further clarify the role of innate lymphoid cells in CRS pathogenesis. Furthermore, one limitation of this study is the potential interdependence between the quantitative expression of PBMCs and tissue-based modulation of various markers. Changes in the expression of specific markers within tissues may be reflected in the composition, activation status, or gene expression profiles of peripheral blood mononuclear cells (PBMCs). Conversely, systemic immune responses represented by PBMCs may correlate with local tissue alterations [25]. However, innate lymphoid cells (ILCs) are predominantly tissue-resident immune cells enriched at barrier tissues and are only sparsely represented in peripheral blood, thereby limiting the extent to which PBMC analyses can accurately reflect tissue-specific immune alterations. Furthermore, due to their very low frequency in peripheral blood, functional analyses of ILCs are technically challenging and often difficult to interpret in a robust and reproducible manner. Consequently, the transferability of peripheral blood findings to local tissue-specific immune processes remains limited [26].

## 5. Conclusions

NK cell frequencies in CRSsNP tissue are comparable to those in peripheral blood and higher than those in CRSwNP tissue, indicating a stronger involvement of NK cells in CRSsNP-associated inflammation. Tissue-resident NK cells in both CRS phenotypes display a more heterogeneous and less mature phenotype than circulating NK cells, characterized by broader CD16 expression and increased proportions of CD56^dim^CD16^dim^/^−^ cells. In CRSwNP, elevated GATA3 expression in polyp-derived ILC2s reflects a dominant type 2 inflammatory environment. Increased TCF1/7 expression in polyp NK cells further indicates a less mature NK cell population in CRSwNP compared with that in CRSsNP. Distinct IL18Rα expression patterns across cell subsets highlight differences in local inflammatory signaling between CRS phenotypes.

## Figures and Tables

**Table 1:** Patient characteristics.

	CRSwNP (*n* = 11)	CRSsNP (*n* = 10)	Control (*n* = 7)
Asthma (*n*)	4	2	0
Sex (f:m)	3:8	6:4	4:3
Previous surgery (*n*)	6	3	1
Age (years)	47 ± 19	51 ± 30	40 ± 23
Allergies	House dust mites (2)	Pollen + grasses (1)	
	Pollen + house dust mites (2) NERD (1) NERD + house dust mites (1) Ash tree (1)	Early bloomers + house dust mites (1) Grasses (1) House dust mites (1) Cortisone (1)	

**Table 2:** Key immunological markers in tissue-resident innate lymphoid cells in CRSwNP and CRSsNP.

Cell Population	Marker	CRSwNP (polyp/mucosa)	CRSsNP (mucosa)
NK cells	Frequency among lymphocytes (%)	7.59/9.94	11.04
NK cells	CD16^+^ NK cells (%)	35.81/30.78	17.02
NK cells	TCF1/7 expression (rMFI)	8.73/8.44	6.46
ILC2	GATA3 expression (rMFI)	7.91/4.99	5.48
ILC3	Nkp44^−^ subset (%)	80.89/91.69	70.64

Abbreviations: CRSwNP—chronic rhinosinusitis with nasal polyps; CRSsNP—chronic rhinosinusitis without nasal polyps; ILC—innate lymphoid cell; NK—natural killer cell; rMFI—relative mean fluorescence intensity.

## Data Availability

The data presented in this study are available on request from the corresponding author.

## Abbreviations

The following abbreviations are used in this manuscript:

ILCs	Innate lymphoid cells
NK	Natural killer
CRS	Chronic rhinosinusitis
CRSwNP	Chronic rhinosinusitis with nasal polyps
CRSsNP	Chronic rhinosinusitis without nasal polyps
